# Empowering patients through eHealth: a case report of a pan-European project

**DOI:** 10.1186/s12913-015-0983-0

**Published:** 2015-08-05

**Authors:** Emanuele Lettieri, Lia P. Fumagalli, Giovanni Radaelli, Paolo Bertele’, Jess Vogt, Reinhard Hammerschmidt, Juan L. Lara, Ana Carriazo, Cristina Masella

**Affiliations:** Department of Management, Economics and Industrial Engineering, Politecnico di Milano, 4/B Lambruschini Street, Milan, 20156 Italy; Warwick Business, University of Warwick, CV4 7AL Coventry, UK; Empirica Gesellschaft für Kommunikations- und Technologieforschung mbH, 2 Oxfordstr, Bonn, D-53111 Germany; Fundación Pública Andaluza Progreso y Salud (FPS), 13 Avda. Américo Vespucio, Sevilla, 41071 Spain; Andalusian Health Service/Regional Ministry of Equality, Health and Social Policies of Andalusia, 14 Av. Hytasa, Sevilla, 41071 Spain

## Abstract

**Background:**

This paper crystallises the experience developed by the pan-European PALANTE Consortium in dealing with the generation of relevant evidence from heterogeneous eHealth services for patient empowerment in nine European Regions. The European Commission (EC) recently funded a number of pan-European eHealth projects aimed at empowering European patients/citizens thus transforming the traditional patient/citizen role in the management of their health (*e.g.*, PALANTE, SUSTAIN, CARRE, HeartCycle, Empower). However, the heterogeneity of the healthcare systems, of the implemented services and of the target patients, the use of *ad-hoc* definitions of the salient concepts and the development of small-size experiences have prevented the dissemination of “global” results and the development of cumulative knowledge. The main challenge has been the generation of large-scale evidence from heterogeneous small-size experiences.

**Discussion:**

Three lessons have been collectively learnt during the development of the PALANTE project, which involves 9 sites that have implemented different eHealth services for empowering different typologies of patients. These lessons have been refined progressively through project meetings, reviews with the EC Project Officer and Reviewers. The paper illustrates the ten steps followed to develop the three lessons.

The first lesson learnt is about how EC-funded projects should develop cumulative knowledge by avoiding self-crafted measures of outcome and by adopting literature-grounded definitions and scales. The second lesson learnt is about how EC-funded projects should identify ambitious, cross-pilot policy and research questions that allow pooling of data from across heterogeneous experiences even if a multi-centre study design was not agreed before. The third lesson learnt is about how EC-funded projects should open their collections of data and make them freely-accessible to the scientific community shortly after the conclusion of the project in order to guarantee the replicability of results and conclusions.

**Summary:**

The three lessons might provide original elements for fuelling the ongoing debate about the capability of the EC to develop evidence-based policies by pooling evidence from heterogeneous, local experiences.

## Background

Patient empowerment can be defined as the acquisition of motivations and abilities that patients might use to improve the participation in decision-making, and thus improve their power in their relationship with professionals [1]. Patient Empowerment has become a key priority for policy-makers and professionals, who assume that it will increase the sustainability of the present paradigms of care delivery [2]. Coherently, many initiatives have been implemented in the last years for empowering patients in self-management [3] and shared decision-making [4]. Several providers, in particular, have pursued eHealth solutions for patient empowerment, *i.e.,* the delivery or enhancement of health care services or health care information through the Internet and related technologies [5]. eHealth might contribute to radically rethink the patient-professional relationship because it overcomes spatial and temporal constraints, develops home-based solutions integrated within the National/Regional Healthcare Systems, integrates different functionalities, *e.g.,* information, education, and communication [6–9]. Following this premise, the European Commission (EC) recently funded a number of pan-European eHealth projects aimed at empowering European patients/citizens to take a more active role in the management of their health (*e.g.*, PALANTE, SUSTAIN, CARRE, HeartCycle, Empower).

Despite such wealth of experiences, however, it is still difficult to build a consistent story on the role that eHealth might play in empowering patients [10, 11], primarily because the concept of “patient empowerment” remains ambiguous [1], and has acquired over time multiple meanings for professionals [12, 13]. Psychologists, hospital managers, nurses have promoted their specific views on what empowerment is and how it might be achieved, limiting the possibility to reach a shared understanding of this concept and agreement on its practical development. The heterogeneity of patients and of the social contexts of European Countries have added further complexity, leading to the implementation of several small scale, country-specific eHealth-based services. Their harmonisation is now a policy priority to enhance the dissemination of local knowledge and experiences on patient empowerment and avoid the upcoming projects constantly ‘reinvent the wheel’.

Large-scale, nation-wide eHealth-based services for patient empowerment cannot be regarded the desired “silver-bullet” solutions, since previous attempts like NPfit or the Whole System Demonstrator have failed to recognise and develop local diversities. Similarly, previous EC-wide projects on patient empowerment stumbled because of three main limitations. First, they have adopted heterogeneous and self-crafted definitions of patient empowerment, which limited the possibility to compare local experiences and learn from a common pool of knowledge. Second, they have collected small-size and pilot-specific data on outcomes, costs, satisfaction or access, struggling to develop acceptable levels of evidence. Third, scales employed to measure significant constructs―such as patient empowerment, ease of use, perceived value―are self-crafted by each project team, more intended for local impact assessment exercises, than for the accumulation and dissemination of knowledge in the scientific community. As a result, a lot of potentially valuable evidence about the role of eHealth solutions to empower patients has struggled to inform evidence-based policies from the EC. Considering these limitation, we thus ask: in the context of EC project involving different Countries and organisations, how can we knowledge development and sharing be organised to allow the comparability of results and facilitate the translation of local experiences in EC-wide policies?

To address this question, we report the experience of PALANTE (**PA**tients **L**eading and m**AN**aging their heal**T**hcare through **E**health) project, which involves 9 sites (namely, “pilots”), each of which has implemented a different eHealth service for empowering different typologies of patients (cf. the next sections). This heterogeneity has facilitated the cross-fertilisation among different eHealth experiences and generation of collective knowledge, but has also the Consortium to many of the limitations described above.

Reflecting on the experience of the PALANTE Consortium, the paper develops three lessons regarding the possibility to pool data from 9 diverse pilots and inform the EC about the role of eHealth solutions in empowering patients.

This paper is organised as follows. The next section briefly describes the methods implemented by the PALANTE Consortium to collect high-quality evidence and the case study. Then, the main results are displayed. The last section summarises the main lessons of this study that will be confirmed with the completion of the project evaluation.

## Discussion

### Study design approach

This section details the phases undertaken to design an impact assessment methodology that matches the need for a robust research design with the contingencies of the 9 eHealth services implemented, in terms of normative constraints, available resources, own goals, maturity of the eHealth service. This research is not based on patients’ clinical data, but on discussions held during the meetings with Consortium partners, the EC Project Officer and three EC reviewers (two academicians from Information Science and one senior manager from a leading, global IT company). All PALANTE pilots adhered to the European and national legislations (*e.g.,* EC Directive 46/95 on data protection) protecting privacy, security and ethical issues and obtained the authorisation, when needed, from the related ethical Committees.

The lessons learnt that will be illustrated later emerged from a journey that the PALANTE Consortium undertook along ten phases in 42 months (lifetime of the project).

First, an urgent and relevant policy and research question was agreed with the nine pilots; namely providing EC decision-makers with evidence on how eHealth contributes to patient empowerment. This clear, synthetic goal was the product of several one-to-one and collective teleconferences and was formalised in a collective vis-à-vis meeting with all partners during the first progress meeting held in June 2012 in Milan (Italy).

Second, the Consortium reviewed a list of etymologically similar concepts (*e.g.*, patient empowerment, engagement, activation, involvement, participation, enablement) to avoid uncertainty among pilots about the main endpoint of their activities and in further dissemination activities. This choice has been informed by the results of a systematic literature review carried out by Politecnico di Milano as academic partner of the PALANTE project. Methods and results of this review are available in another manuscript [1]. Based on the results of the systematic literature, the PALANTE Consortium adopted the concept of “patient activation” and its Patient Activation Measure (PAM) scale [14] to operationalise the broad concept of “patient empowerment”. An *activated patient* knows how to manage her condition, maintain functioning and prevent health declines; she has the skills and behavioural repertoire to manage her condition, collaborate with her health providers, maintain her health functioning and access appropriate and high-quality care [14]. This concept was found more suitable to explain and represent the main purposes of the eHealth services implemented by the 9 pilots.

Third, the significant heterogeneity of the eHealth services discouraged an immediate direct comparison (Table 1). This limitation was overcome by deconstructing the services into a combination of three basic functionalities (*e.g.*, information, education, communication). It was then possible to compare the 9 implemented services in terms of their basic functionalities. Additionally, data collected at the pilot level could be pooled according to the specific functionality. As result, the goal of the PALANTE project has been reframed as providing evidence on if and how eHealth-enabled information, education and communication might contribute to a positive/negative variation in patient activation. To our best knowledge, this is an original solution to data pooling in multi-centre studies on eHealth-based care delivery.

**Table 1:** Pilots’ features. Shows the heterogeneity in terms of main goals, services offered, target patients, and size of the 9 pilots involved within the PALANTE project

Pilot #	Main services offered	Target users
Pilot 1 – Andalusia (Spain)	*Objective.* To empower patients with diabetes and improve quality of life and diabetes management through a new eHealth solution that comprises:	Diabetes 7,000 patients *6,500 Type 2* *500 Type 1*
	▪ Patient access to Personal Health Record and management of personal health information (medications, appointments, exams, allergies, clinical reports)	
	▪ Chronic disease management support services: Recording and tracking of patient’s own measurements (blood sugar levels, weight, blood pressure) through tensiometers and glucometers	
	▪ Tailored education and lifestyle guidance through (virtual) games	
	▪ Secure messaging between patients and health care professionals	
Pilot 2 – Lombardy (Italy)	*Objective.* to empower patients with chronic heart failure disease by involving them in and integrate care pathways where a specialist follows them.	Chronic Heart Failure Disease 3,400 patients
	This is done through the SISS-DCPA tool available for producing Clinical Care Path document at the hospital, publishing and versioning on the EHR to share it with GP and patient. It comprises:	
	▪ Patient access to Personal Health Record and management of personal information: citizens can see his/her clinical documents and upload documents	
	▪ Tailored education and lifestyle guidance (guidelines, recommendations on lifestyle to adopt, diet, physical activities to do)	
	▪ Timeline view providing bird’s eye view of the HER	
	▪ OTP access (OTP sent via Mob. Phone) & Access with mobile devices	
	▪ Better integration with new services of booking and payment	
Pilot 3 – Turkey	*Objective.* To empower patients that suffer from ankylosing spondylitis, by follow them through a service that permits to have:	Ankylosing Spondylitis 2,000 patients
	▪ Tailored education and lifestyle guidance through Educational exercise videos on disease and treatments	
	▪ Messaging service between patient and doctor	
	▪ Diary service which allows patients to record their daily or weekly health status and inform the doctors if necessary (Patient Diary)	
	▪ Remote follow-up	
	▪ Chronic disease management	
	▪ Remote consultation	
	▪ Medication and non-pharmacological treatment follow-up and medication and non-pharmacological treatment benefit analysis	
	▪ Reminders	
Pilot 4 – Norway	*Objective*. To empower patients with diabetes and hospitalised patients for any disease by providing the following eHealth services:	Diabetes 1,550 patients Discharge Notes 1,3 million of patients
	▪ Electronic discharge notes in the local electronic health record	
	▪ Modules tailored for chronically ill patients (diabetes)	
	▪ Consultation form and self-reporting tools to support self-management	
	▪ A guidance tool that helps visualizing factors (food, insulin and physical activity) that influence blood sugar levels in a 24 hour clock, also used as a communicational tool	
	▪ Recording and tracking of patient’s own measurements (blood sugar levels, weight, blood pressure, HbA1c)	
	▪ Patient access to Personal Health Record	
	▪ Secure messaging between patients and health care professionals	
	▪ Integrated with MinJournal (Patient Portal)	
Pilot 5 – Styria (Austria)	*Objective*. To empower patients on their condition of X-ray exposure for estimating the risk level in making a new exam through an eHealth services that comprises:	X-ray exposure 1,183 patients
	▪ Patient access to his/her personal eX-ray-Record that reports personal achieved level of X-ray exposure	
	▪ Storing of individual information about radiation exposure data for all examinations into the existing Hospital Information System (HIS)	
	▪ Develop an informative and comprehensible presentation for the radiation exposure data	
	▪ Provide access for patients to their personal eX-ray-Record via a patient-portal on the internet (and for health professionals via the HIS)	
Pilot 6 – Czech Rep.	*Objective.* To empower parents in taking care of their children through a eHealth service that permits to:	Children health management 3,352 patients
	▪ Scheduling appointments	
	▪ Having access to Personal Health Record	
	▪ Monitoring individual parameters related to children growth	
	▪ Accessing to the Vaccinations program	
	▪ Monitoring the Preventive check-outs calendar	
	▪ Monitoring the child growth (height/weight)	
	▪ Sending alert reminders via SMS	
Pilot 7 – Basque Country (Spain)	*Objective*. To empower patients with COPD by including them in a telemedicine program with educational module to follow. The eHealth service includes:	Asthma & COPD 150 patients
	▪ Integration of all the data and information that a patient generates through the health services (health habits, treatment, symptoms, etc.)	
	▪ Patient access to Personal Health Record	
	▪ Tailored education and lifestyle guidance	
	▪ Telecare, Monitoring and Chronic disease management support services	
	▪ Real time health information uploading	
	▪ Secure text & voice messaging and videoconference with the healthcare team and alerts	
	▪ Telerehabilitation (physical exercises)	
	▪ Multi-platform: TV/Kinect & mobile	
Pilot 8 – France	*Objective.* To empower citizens providing the access to the personal information through the national health portal where it is possible:	All Citizens/ Patients
	▪ To view data and to be informed about new documents	
	▪ To correspond securely with a healthcare professional, after his consent	
	▪ To enter information: self-monitoring results, their wishes regarding arrangements at the end of their lives, etc.	
	▪ To download all or some of contents of the personal health folder	
Pilot 9 Denmark	*Objective.* To empower citizens providing the access to the personal information through the national Advanced eHealth portal with the following services:	All Citizens/ Patients
	▪ Patient management of personal health information	
	▪ Patient access to Personal Health Record	
	▪ Chronic disease management support services	
	▪ Lifestyle guidance	
	▪ National web-solution for citizens/patients and health professionals, integrating data from app. 100 sources	
	▪ Online EHRs from hospitals, cross-sectorial personal electronic medicine profile, access to lab/test results	
	▪ List of contacts with public hospitals and publically subsidised contacts in PHC	
	▪ Organ Donor Registration and Living Will	
	▪ Patient’s audit of any access to his/her data	

Fourth, patients’ willingness to engage with the eHealth-enabled functionalities was measured, consistently with the well-established Technology Acceptance Model (TAM) [15], through patients’ perceived value and ease of use.

Fifth, a sample of control variables was identified from the literature to control potential confounding factors and gather better explanations of results.

Sixth, the theoretical framework linking PAM-related and control variables to TAM-related outcomes was discussed with all pilots in order to collect potential criticalities (*e.g.*, in the case of Norway the legal framework does not allow identification of the respondent because of privacy concerns, and this inhibited a longitudinal research design).

Seventh, a very detailed protocol for data collection was produced and pilots were trained on its strict application to assure data pooling and significant evidence generation.

Eighth, the research design was discussed during the first review meeting with three reviewers appointed by the EC and the Project Officer in April 2013 in Brussels. The methodology was approved, being regarded capable (i) to generate significant evidence through data pooling from a multi-centre pan-European project, (ii) to be translated to other projects about eHealth and patient activation, and (iii) to be methodologically sound within the literature and academic standards.

Ninth, three lessons learnt about how EC-funded projects could behave to generate significant evidence were crystallised during the fifth progress meeting in September 2014 in Istanbul. Project Officer and reviewers provided the PALANTE Consortium with the recommendation to produce lessons which could be shared with other EC-funded projects. The three lessons were agreed by all partners within the Consortium.

Tenth, the lessons were shared in an open conference organised by the PALANTE Consortium in June 2015 in Brussels. Feedbacks collected from attendees―academicians as well as practitioners―allowed the refinement and improvement of the lessons.

### Case study

Table 1 shows the heterogeneity in terms of main goals, services offered, target patients, and size of the 9 pilots involved within the PALANTE project.

Such heterogeneity was intentional, since the Consortium sought the opportunity to collect insights from very different experiences and provide the EC with diverse narratives on how eHealth could be used to activate patients. Pilots were then selected to account for different types of disease, levels of interaction between patients and healthcare professionals and types and innovativeness of eHealth-enabled solutions. The selection of polar-extremes cases [16] generated local expectation of pilot-specific impact assessment exercises, with the consequence of limiting any generalizability of results. This kind of expectation is common in EC-funded project since pilots are more interested in gathering an in-depth understanding of the pros and cons of their eHealth service rather than formalise more theoretical lessons from the comparison of heterogeneous experiences. This is particularly true when the conduction of a multi-centre study has not been agreed before. In this regard, we discussed with pilots the opportunity of gathering more robust evidence by pooling their data through the design of an *ad-hoc* impact assessment framework.

In order to counter such expectations, address pilots’ heterogeneity and guarantee the collection of high-quality, larger-size evidence, three challenges (and consequent lessons learnt) arose in the Consortium:

- The crystallisation of the main concept/phenomenon under observation (*i.e.*, patient activation) and of the main explanatory variables (*i.e.*, ease of use and perceived value);
- The development of an *ad-hoc* research design for the impact assessment that allows data pooling from heterogeneous pilots;
- The delivery of original, valuable, timely and robust analyses of collected data within a very constrained time span.

Finally, all these challenges had to be faced through the implementation of generally accepted academic standards in order to facilitate both the dissemination of results and the legitimacy of EC’s policy-making [17].

Henceforth, we detail the three main challenges and how they have been tackled by the PALANTE Consortium*.*

### Crystallisation of the main concept/phenomenon and explanatory variables

In the first project meeting, it emerged clearly that the concept of “patient empowerment” had different meanings for the people sitting at the table. This ambiguity limited the possibility to “speak the same language” both inside and outside the project (*e.g.*, with the SUSTAINS Consortium that was working on patient empowerment through the access to and use of Electronic Medical Records), in particular with healthcare professionals. The same concern emerged with respect to the explanatory variables. While all pilots agreed that concepts such as user-friendliness of the eHealth service, patient’s perception that pros outweigh cons, patient’s self-efficacy with the eHealth service would have been salient in predicting the success of any eHealth-enabled care delivery, there was not an alignment on the metrics for their measurement and analysis. Each pilot had instead an individual list of metrics employed in past EC-funded or publicly funded projects, with unclear scientific foundations.

The Consortium addressed this issue reviewing past contributions on eHealth-enabled solutions for care delivery and on patient empowerment. This allowed furthering the discussion with the pilots and positioning the expected results according to past research [18].

On the one hand, past studies agreed that in order to take advantage of an eHealth-enabled solution for care delivery, patients must be *willing* and *able* to interact with the new system (*e.g.,* personal health record and secure messaging [19], eHealth service [20]). This argument underlines the importance of the patients’ perception of the system that has to be used. This perception is influenced by two main elements, *i.e.,* the patient should expect to reach some benefits thanks to the use of that system; and to use the system without difficulty. Both elements can be estimated through the TAM scale [15], which was developed to predict and evaluate users’ acceptance of different information technologies. The scale includes the construct of usefulness, which represents “the degree to which a person believes that using a particular system would enhance his or her job performance” [21]; and ease of use, which represents “the degree to which a person believes that using a particular system would be free of effort” [21]. The literature also suggested relevant control variables to be taken into account for possible confounding factors, *i.e.,* gender, age, education level, ICT experience, experience with disease and trust in regional/national healthcare system [22–24].

On the other hand, the literature did not provide the Consortium with a straightforward solution to operationalise patient empowerment, since this concept is still under discussion and has important overlaps with neighbour concepts―such as activation, involvement engagement, enablement, participation. By carrying out a systematic literature review [1], the Consortium addressed this issue selecting the concept of “patient activation” [14] to operationalise “patient empowerment” and to position the expected results within the ongoing international debate.

### Data pooling from heterogeneous pilots

A second core issue related to the capability to pool data from very different pilots. As shown in Table 1, pilots were implementing significantly different eHealth-enabled solutions, which inhibited a direct comparability of results. During the first progress meeting in Milan (June 2012), pilots discussed the pros and cons of designing a multi-centre study in such a context. Pilots claimed that their heterogeneity required pilot-specific impact assessment exercises in order to take into account the contingencies of each pilot. Furthermore, their previous experiences with EC-funded projects suggested that small-sise, pilot-specific impact assessment exercises were more efficient and valuable for local dissemination. On the other hand, the literature did not offer examples of how to cope with this heterogeneity since past studies compared and pooled data from similar eHealth services (*e.g.*, telemonitoring [25], Electronic Medical Records [26, 27]).

The Consortium regarded these arguments as an opportunity to develop an innovative approach that could be valuable, for instance, to all projects that receive public funds and share the same heterogeneity, but still has to gather evidence that might matter at the EC-level. The most practical solution for a common assessment is deconstructing each eHealth-based service according to its basic functionalities since all services seek to activate patients by informing, educating and communicating with them. Specifically, *information* refers to all kind of information shared through the eHealth service, such as alerts and notifications about treatments, reminders on appointments, opportunities to book medical visits or other health services, exchanges of personal health information or documents. *Education* refers instead to all aspects that affect the education of patients, who learn by using devices and educational materials (video, suggestions, documents, care plan), learn the meaning of their health results, how to increase their skills and to manage their own diseases on a daily basis. Finally, *communication* refers to all kind of communication between healthcare providers and patients (*e.g.*, texts, video or e-mail, calls).

In this view, the eHealth solutions implemented by the pilots have been conceptualised as an organised, coherent bundle of these three functionalities (Table 2).

**Table 2:** Deconstruction of pilots’ eHealth service in its main functionalities. Reports the organization of the eHealth solutions implemented by the 9 pilots according with the three functionalities identified: information, education and communication

Pilot #	eHealth functionalities		
	Information	Education	Communication
Pilot 1 – Andalusia (Spain)	● Patient access to Personal Health Record and management of personal health information (medications, appointments, exams, allergies, clinical reports)	● Tailored education and lifestyle guidance through (virtual) games: Diabetes self-management Education (diet counseling, daily activities, measurements, games)	● Communication with their healthcare team: Secure messaging between patients and health care professionals
	Chronic disease management support services: Recording and tracking of patient’s own measurements (blood sugar levels, weight, blood pressure) through tensiometers and glucometers		
Pilot 2 – Lombardy (Italy)	● Patient access to Personal Health Record and management of personal information: citizens can see his/her clinical documents and upload documents	● Tailored education and lifestyle guidance (guidelines, recommendations on lifestyle to adopt, diet, physical activities to do)	
	● Access to Integrated Care Pathways (for healthcare professionals and patients) and data entry and updates (only for healthcare professionals)		
	● Timeline view providing bird’s eye view of the HER		
	● Better integration with new services of booking and payment		
	● Patient summary and structuration		
Pilot 3 – Turkey	● Access to Personal Health Record and Electronic Health Record	● Tailored education and lifestyle guidance through Educational exercise videos on disease and treatment	● Messaging service between patient and doctor
	● Chronic disease management support service		
	● Diary service which allows patients to record their daily or weekly health status and inform the doctors if necessary (Patient diary)		
	● Remote follow-up		
	● Remote consultation		
	● Medication and non-pharmacological treatment follow-up and Medication and non-pharmacological treatment benefit analysis		
	● Reminders		
Pilot 4 – Norway	● Electronic discharge notes in the local electronic health record	● A guidance tool that helps visualizing factors (food, insulin and physical activity) that influence blood sugar levels in a 24 hour clock	● Consultation form and self-reporting tools to support self-management Internet-based patient/ provider communication and interaction
	● Modules tailored for chronically ill patients (diabetes)		Secure messaging between patients and health care professionals
	● Recording and tracking of patient’s own measurements (blood sugar levels, weight, blood pressure, HbA1c)		
	Patient access to Personal Health Record		
Pilot 5 – Styria (Austria)	● Patient access to his/her personal X-ray-Record that reports X-ray exposure		
	● Storing of individual information about radiation exposure data for all examinations into the existing hospital information system (HIS)		
	● Develop an informative and comprehensible presentation for the radiation exposure data		
	Provide access for patients to their personal eXrayRecord via a patient-portal on the internet (and for health professionals via the HIS)		
Pilot 6 – Czech Republic	● Scheduling appointments		● Sending alert reminders via SMS
	● Patient access to Personal Health Record		
	● Monitoring individual parameters related to children growth		
	● Accessing to the Vaccinations program		
	● Monitoring the Preventive check-outs calendar		
	● Monitoring the child growth (height/weight)		
Pilot 7 – Basque Country (Spain)	● Integration of all the data and information that a patient generates through the health services (health habits, treatment, symptoms…)	● Tailored education and lifestyle guidance	● Secure text & voice messaging and videoconference with the healthcare team and alerts:Call/videoconferencing/audio and text messaging; the patient can be in contact with other people in charge of his/her care (family, care assistants, etc.), Sharing/discussing with other professionals about certain symptoms, treatments, etc.
	● Patient access to Personal Health Record		
		Telerehabilitation (physical exercises)	
Pilot 8 – France	● Patient access to personal heath folder and download of personal contents	● Tailored education and lifestyle guidance	● To correspond securely with a healthcare professional, after his consent
	● To view data and to be informed about new documents	● Telerehabilitation (physical exercises)	
	● Data entry: self-monitoring results, their wishes regarding arrangements at the end of their lives, etc.		
	● To download all or some of contents of the personal health folder		
Pilot 9 – Denmark	● Patient management of personal health information		
	● Patient access to Personal Health Record		
	● Chronic disease management support services		
	● Online EHRs from hospitals, cross-sectorial personal electronic medicine profile, access to lab/test results		
	● List of contacts with public hospitals and publically subsidized contacts in PHC		
	● Organ Donor Registration and Living Will		
	● Patient’s audit of any access to his/her data		

Observing the role that eHealth *functionalities* might play in enabling patient activation, the PALANTE Consortium achieved two benefits. On the one hand, data collected from heterogeneous pilots could be pooled together into a common dataset. This increased the capability to generate reliable evidence that might be of value for the EC to ground its policy on the promotion of eHealth solutions for patient activation. This solution was of paramount relevance because it showed that more fine-grained observation of functionalities can overcome the shortcomings of the traditional pilot-constrained impact assessment. Furthermore, an analysis based on functionalities will provide the opportunity to disentangle their impact on patient activation and to understand which functionalities―or combinations of them―are more likely to positively activate patients. This knowledge can provide researchers, policy-makers, professionals, suppliers with more detailed guidelines for the development of cost-effective eHealth solutions for patient activation.

### Delivery of the analyses within a very constrained time span

The PALANTE project has a lifespan of 42 months. Within this timeframe, only the last six months could be dedicated to data analysis. This situation is very likely to happen in many EC-funded projects since the development/implementation of the eHealth-enabled solutions as well as data collection require significant portions of the whole project lifespan. As result, at least two shortcomings arise with respect to the exploitation of the huge mass of data collected. On the one hand, the PALANTE Consortium will have to focus its attention on the analyses agreed with the EC at the beginning of the project, when it is not possible to anticipate all peculiarities, issues and variables that could play a role within the pilots. The risk is not to include possible relevant analyses which are not formally signed with the EC. For example, while analyses could be carried out to verify if the three eHealth-enabled functionalities affect patient activation with respect to the set of pooled data, questions arose about the possibility to perform pilot-specific analyses. On the other hand, collected data could be investigated only by partners of the PALANTE Consortium, losing the opportunity to collect points of view of other experts that could identify and suggest new way to explore data. This has two specific main limitations. First, the capability to read these data from different, original angles was affected. If other researchers, professionals, policy-makers can access these data they could probably bring new research and policy questions, enabling the generation of additional knowledge. Second, opening the dataset to others significant stakeholders would guarantee the reliability of results because it would allow the replicability of all analyses. This would reinforce the strength of the final policy recommendations, so that the EC would be justified in leveraging on these results and orienting future research and projects. The growing concerns about the quality of the evidence that underpins healthcare decision-making [28–32] obliges more than ever EC-funded projects―being publicly funded by taxpayers―to consider the possibility to open their datasets to the scientific community in order to guarantee the reliability of their results. In this regard, the EC has already moved the first steps in this direction, giving the opportunity to winners of H2020 grants to apply to the open data protocol (http://ec.europa.eu/digital-agenda/en/open-data-0). This initiative needs to be treated with attention in order to manage sensitive aspects like privacy that involves the laws of different Countries. Making data sets publicly available is a delicate matter, particularly when the data set is sourced from a wide-range of member states. Legal concerns regarding confidentiality of patient data and permission requirements need to be well examined within the state legislation for each member state involved. Additionally, rules on using the data for publication and the process of gaining consent from the data’s source also need to be taken into consideration; particularly, when the data yields a negative result which may make its use politically sensitive.

Such concerns are being taken into consideration by the PALANTE Consortium as it required the definition and submission of informed consent documents to patients at the enrolment in the project, and it is still discussing methods for maintaining and making available the dataset following the conclusion of the project.

A chief concern of the Consortium is considering the opportunity for further analyses post-PALANTE lifetime, which would generate additional value for the EC as well as for all healthcare eco-system. A first step towards this goal is sharing data within the Consortium to increase the transparency of methods and results with partners and improve the explanation of the results. This internal sharing and opening of the dataset is also an opportunity to learn and understand potential issues that can arise and that require to be treated increasing the knowledge needed for managing future external access to the dataset.

## Results

At the moment the PALANTE project is in progress. The administration of the baseline survey has been completed and the collected data are under analysis. A second survey is currently in the field to gather data on patients’ experience after six months of service use. While it is not yet known whether the eHealth functionalities positively affect patient activation, the PALANTE Consortium argues that the solutions implemented for its assessment might *per se* offer an original, valuable and viable way to overcome the limitations of the traditional generation of evidence in EC projects and, more generally to publicly-funded inter-national projects. The EC needs to ground its policy-making on significant―in terms of size and robustness―sources of evidence that come from both local and pan-European projects. Furthermore, European Regions look to such research projects for reliable and pertinent evidence when making development decisions for their healthcare systems. To support this, we have formalised three lessons that might overcome the shortcomings of small-scale, pilot-specific evaluations. These lessons have policy implications interesting for the EC and offer new insights to further the debate about the implementation of evidence-based policies in healthcare.

The first lesson learnt is the “*relevance of developing cumulative knowledge*”. Any EC-funded project should adopt clear terminologies and scales, following the on-going scientific debates in the literature. In PALANTE project, for instance, patient empowerment was replaced by patient activation as result of a systematic literature review. Investments made to stimulate patients taking a proactive role in their health require the development of a unique, consistent story that could cumulate evidence from various projects. This would be impossible if different terminologies, definitions and measurement scales are adopted. In this regard, these concepts should be taken from past scientific literature in order to guarantee the accumulation of data and knowledge and the creation of significant evidence for the EC. The PALANTE Consortium adopted a consolidated scale―*i.e.,* the PAM scale―that has already been used across a wide variety of past studies. This means that evidence collected by PALANTE will add new insights to what is already known. Whether the application of the same scale to different eHealth solutions and patient cohorts hamper construct validity will be evident in the project’s final results.

The second lesson learnt addresses the “*relevance of developing large-size knowledge*”*.* We suggest that any EC-(or publicly) funded project involving multiple pilots should implement a strategy for a multi-centre research design to pooling local data into a common repository. This strategy is especially critical when pilots are committed to small-size, innovative eHealth solutions ― and suggest the involvement of coordinating actors/organisations that could mediate pilots’ interests. The informative power of pan-European projects can be especially increased through the careful identification of cross-pilot policy questions (*e.g.,* do eHealth-enabled information/education/communication functionalities positively affect patient activation?) and their subsequent formalisation into a literature-based, cross-pilot questionnaire data collection.

The third lesson learnt is the “*relevance of developing open, freely-accessible sources of knowledge*”*.* We argue that any EC-funded project should make its full impact assessment dataset available to the scientific community shortly after the conclusion of the project. This should constitute a formal output of any project. The pros of this policy-choice are evident. Opening the dataset to the scientific community and any key stakeholders (*e.g.,* healthcare professionals, regional officials, hospital managers, patient associations) means they could replicate the main analyses conducted within the project and to implement other studies to the same dataset. The cons refer to the protection of confidential information defended by national and/or European laws; to the capability of pilots and other partners to disseminate the project findings through publications in high-quality journals or in relevant conferences/workshops; to the possibility that non-project participants can profit academically without bearing the huge effort of data collection, and to the potential misuse of data. Finally, pilots have a vested interested in being first on publication based on their data. These issues will be addressed as PALANTE discusses how to provide access to and maintain data sets once the project has concluded and may provide a map for guide future projects when making their data publicly available.

Concluding, the project teams have the responsibility to design methodologies for their impact assessment exercises that are able to meet the recommendations cited above. The involvement within the project Consortium of partners with significant expertise on Health Technology Assessment (HTA) and Evidence-Based Policy-making can help improve the quality of research designs and of gathered evidence.

## Summary

These lessons might be valuable for different stakeholders and could be applied through a number of strategies which this manuscript seeks to facilitate.

First, the EC officers might lever on the PALANTE experience to promote the adoption of more sophisticated methodologies for the impact assessment exercise in other funded projects with the aim of collecting a higher level of evidence. Upstream, the EC officers might change their ranking system for future proposals by allocating higher scores to proposals that take into account the generation of high-quality evidence in terms of size and coherence to the literature. Downstream, the EC officers might orient future research toward implementing facts-based health strategies, rationalizing investments and sifting out unprofitable research fields/directions/initiatives.

Second, scientific/project coordinators of EC-funded projects―as well as professionals which are involved in these projects―might benefit from these lessons because they exemplify real-life situations related to coping with the high complexity of an impact assessment exercise on heterogeneous eHealth services in different Countries.

Third, developers or suppliers of eHealth services might benefit from the PALANTE story in terms of renewed willingness and increased ability to gather high-quality evidence about the capability of their services to actually empower patients. In an European context characterised by shrinking financial and human resources, policy-makers must prioritise which small-size eHealth services should grow to a larger scale and become institutionalised.

Fourth, scholars of Health Policy, Healthcare Management and Information Science might benefit from this study in terms of new insights to further the ongoing debate about the development and implementation of population-based trials. The significant costs―in terms of money and time―of traditional randomised clinical trials obliges the exploration of other pathways to gather high-quality evidence to support policy-making. In this regard, the development of embedded solutions within eHealth services to collect data could offer an interesting and methodologically sound alternative.

## Abbreviation

EC: European Commission
PALANTE: **Pa**tients **L**eading and m**AN**aging their heal**T**hcare through **E**health
HTA: Health Technology Assessment
PAM: Patient Activation Measure
TAM: Technology Assessment Model
ICT: Information and Communication Technology
